# Utilization of sexual and reproductive health services among young people living with HIV and attending selected HIV clinics in selected sub-counties of Nairobi, Kenya

**DOI:** 10.12688/openreseurope.17611.2

**Published:** 2025-07-25

**Authors:** Nomsa Phiri, Susan Mambo, Careena Otieno Odawa

**Affiliations:** 1 Department of Environmental Health and Disease Control, Jomo Kenyatta University of Agriculture and Technology, Nairobi, Nairobi County, Kenya; 2 Department of Community Health, Great Lakes University of Kisumu, Kisumu, Kisumu County, Kenya

**Keywords:** HIV, sexual reproductive health, youth, adolescent, health services

## Abstract

**Background:**

Young people living with HIV in Sub-Saharan Africa account for the largest proportion of the vulnerable population in the world. Kenya has little evidence to showcase the utilization of sexual and reproductive health services among young people living with HIV. Nairobi County has one of the highest HIV burdens among adolescents and youth in the country. Consequently, this study aimed to assess factors associated with the utilization of sexual and reproductive health services among young people aged 15–24 years living with HIV.

**Methods:**

A health facility-based cross-sectional study design with convergent parallel mixed methods technique was used. Purposive sampling with predetermined criteria was used to select six high-volume public health facilities in six high-burden sub-counties of Nairobi. A total of 253 participants completed the semi-structured questionnaires on utilization and associated factors.12 healthcare workers were purposively selected as key informant sessions in individual key informant sessions on their perception of young people’s utilization.

**Results:**

4Stepwise multivariable binary logistic regression was used to analyse the quantitative data using Stata version 14. NVivo software was used to code and thematically analyse the data. Results 47 % of the participants had utilized the services. Collection of condoms (45.7%) was the most utilized while treatment of sexually transmitted infections (8.2%) was the least utilized services. Female sex (AOR--adjusted odds ratio : 3.60 95%, Cl: 1.67-6.40), increase in age (AOR: 2.27 95%, Cl: 1.1C-4.65), HIV status disclosure to a sexual partner (AOR: 2.00 95%, Cl: 1.11-3.80) and privacy for sexual and reproductive health services at a health facility (AOR: 3.27 95%Cl: 1.42-7.60) were factors significantly associated with utilization.

**Conclusions:**

Given higher utilization rates of sexual reproductive services were demonstrated among females and those who disclosed their HIV status to sexual partner , strengthening behavioral interventions to engage young men and disclosure support strategies can address the existing disparities.

## Introduction

More than 50% of young people aged 15–24 globally have unmet needs in sexual and reproductive health yet more than 4 million of this population are living with HIV ( Stackpool-Moore *et al*., 2017). Young people living with HIV in Sub-Saharan Africa account for the largest proportion of the vulnerable population in the world ( UNAIDS, 2020). The rate of increase in poor sexual reproductive health outcomes experienced by young people in sub–Saharan Africa is quite concerningly high ( Odo *et al*., 2018). The high incidence of poor sexual and reproductive health outcomes among the youth is due to the underutilization of sexual and reproductive health services ( Erhabor *et al*., 2013). Utilization can be defined as the extent to which people are making use of the sexual reproductive health services that are already available in the community. Assessment of utilization enables earlier identification of priority areas and designing interventions tailored to people’s needs ( Carrasquillo Chief, 2020). Many literature sources such as the systematic review by ( Belay *et al.*, 2021) demonstrated that utilization was based on the usage of one of the components of the SRH services. The essential package includes counselling, contraception provision, treatment of sexually transmitted infections and counselling ( Belay *et al.*, 2021).

Even though young people in general have been reported to have similar frequencies of sexual activities and shared burden of sociocultural taboo around sex as their peers living with HIV, young people living with HIV face additional unique burden of factors that influence the utilization of these services. Young people living with HIV have distinctive challenges separate from their peers as HIV disclosure dilemma and HIV related stigma and morbidity. The consequences of poor sexual and reproductive health utilization outcomes are more compounded for young people living with HIV compared to their peers. The poor outcomes include an increased risk of sexually transmitted infections, unintended pregnancies and abortions. The complication of poor sexual health is a leading cause of death among young people ( Thongmixay *et al.*, 2019).

 There are a variety of factors that influence the usage of sexual and reproductive services. Individual factors that can influence the uptake of sexual and reproductive services include education level, sexual history family support system, and religious and cultural background. Sociocultural beliefs around young people’s sexuality and gender inequality act as barriers to utilizing sexual and reproductive health services Stackpool-Moore *et al.*, 2017. Health system factors that influence the uptake of sexual and reproductive health services include the attitude of the healthcare staff, communication skills and training of the health staff, and availability of sexual and reproductive health commodities ( Erhabor *et al.*, 2013).

It has been recognized that the creation of a conducive environment leads to more effective health outcomes and interventions ( WHO, 2017). This is why sexual and reproductive health for young people living with HIV has emerged as a global public health concern ( Mkumba *et al.*, 2021). Although there is global awareness of the importance of sexual and reproductive health and its services, there has been difficulty in promoting it, especially in middle- and low-income countries ( Mkumba *et al.*, 2021). There is currently a paucity of data on implementation of monitoring on how services like sexual and reproductive services for young people living with HIV are integrated and utilized in Africa ( WHO, 2019). Kenya is no exception to this challenge.

In response to the global campaign, Kenya has favorable policies that promote sexual and reproductive rights. The policies include the Constitution of Kenya (2010), the National Youth Policy (2007) and the National Adolescent Sexual and Reproductive Health Policy 2016. Kenya has managed to integrate HIV care and treatment services with SRH albeit it varies at different levels of care in the health care sector. There are stand-alone youth-specific centres like DREAM centres that are most highly integrated. Primary health care facilities (level 2) are integrated by default due to the low volume of health care human workforce. For instance, one nurse can run both family planning services and HIV comprehensive care treatment in the same building. This differs in level 3 facilities where the volume of the patient is larger ( National AIDS and STI Control Program, 2020).

Despite the progress in sexual reproductive health, there are still high rates of unintended pregnancies, unsafe abortions and inconsistent condom use among young people in Kenya. Pregnancy is a significant consequence with 18% rate among the young people. This indicates that there is low utilization of sexual reproductive health services ( Center for Reproductive health, 2021). A study done among young people aged 18–24 years in Kenya demonstrated that there is low uptake of reproductive services such as contraception and treatment of sexually transmitted diseases ( Nyang’echi & Osero, 2024).

There is a demonstrated concern about irregularities in accessibility and utilization of youth-friendly services provided in Nairobi and Kenya ( USAID, 2011). Most studies that look at the utilization of sexual reproductive health services tend to focus on the general population of young people. There is a population gap on young people living with HIV in Kenya. A previous cross-sectional study done in Kenya showed that reproductive health knowledge on STIs and contraception methods was inadequate among the youth. The drawback of this study is that the focus on the general population of the youth ( Mbugua & Karonjo, 2018).

Kenya has 131780 young people living with HIV. Nairobi County has one of the highest HIV burdens among adolescents and youth in the country yet this vulnerable subpopulation is underrepresented in sexual reproductive research ( National AIDS Control Council, 2018). There is a paucity of literature examining the utilization of adolescent and youth-friendly services among vulnerable subgroups of the young population as young people living with HIV in Nairobi, Kenya

To fill this gap, primary data collection and analysis were investigated on utilization of sexual and reproductive services among young people living with HIV aged 15–24 in Nairobi County. This paper aims at assessing the determinants of utilization of sexual and reproductive health services among young people living with HIV in Nairobi County. This study expands on the current knowledge base of understudied utilization of sexual and reproductive services among young people living with HIV.

## Methods and materials

### Study design

This was a health facility based cross-sectional study design with mixed method convergent parallel technique that was conducted in Nairobi between 1–30 ^th^ June 2023. The convergent parallel mixed method technique allowed for concurrent collection and analysis of quantitative and qualitative data. This enabled merging the two datasets for comparison in the discussion section.

### Study setting

The study was conducted in Nairobi, the capital city of Kenya. Nairobi is one of the 47 counties of Kenya. It has 17 sub counties. It is situated in the south-central part of the country at an elevation of about 5500 feet ( Kenya National Bureau of Statistics, 2023). It is one of the top counties leading with a high number of young people with HIV. It was estimated to be 24,918 young people living with HIV ( National AIDS Control Council, 2018).

### Sampling technique

The quantitative data was obtained using multistage sampling to select the respondents. Out of 17 sub-counties, there are six high burden HIV sub-counties which are contributing 50% of the total population of young people living with HIV. In each subcounty, a government health facility was selected. Thus, six health facilities were selected. Four of the selected health facilities were level 3. This means they are gazetted as health centres providing comprehensive care according to local needs. One was level two (health dispensary) and the last one was level four (sub-county referral hospital). The criteria for selection included the presence of comprehensive care clinic services (HIV clinics), the volume of the study population attending the clinic and the distribution of the catchment areas involved. Sampling proportional to size was used to determine exact numbers per facility. Furthermore, systematic random sampling was used at the health facility level. A pre-existing register list from the HIV clinics was used to create a sampling frame for all clients who were within the required age range. Since comprehensive clinics are conducted every day for scheduled antiretroviral therapy drug refills, a list of youth attending were sampled. A random starting point on the sampling frame was selected, and consecutive participants were selected at a fixed interval. The sample size for the quantitative arm calculated was 253. Calculations were done using the prevalence of 32.8 % for utilization of sexual reproductive services by a similar study in Ethiopia ( Amaje *et al.*, 2022) at 95% (confidence), Z = 1.96 where p = 0.328 (prevalence) e= 0.05 (the margin of error).

### Participants

The study population comprised young people aged 15–24 years living with HIV, residing in Nairobi and attending HIV clinics. They must provide signed informed consent for those above 18 years or provide written parental consent and assent for those below 18 years. It was also required that participants have a documented HIV positive status. The youth that were mentally incapacitated, were unable to consent and answer questions at the time (critically ill), and had been less than a month in HIV clinic care after HIV diagnosis confirmation were excluded.

In order to gather qualitative data, 12 individual key informant interviews sessions were performed. Purposive sampling techniques were used to select 12 health care providers as key informants that are involved in treatments and managers of the six selected comprehensive care clinics (HIV clinics) in the six selected sub counties.

### Data collection methods and tools

Trained interviewers used pretested semi structured interviews to conduct the face-to-face interviews in the quantitative component. The interviews took place in a private location of the selected HIV clinics and lasted about 30–45 minutes. The interviews were conducted in English and Kiswahili. Four weeks were needed for recruitment and to collect data from participants in the month of June 2023. All young people fitting the criteria were then approached face to face for interviews. Written informed consent was obtained before participation. Informed parental and assent consent was obtained for those who were underage.

The pretested semi structured questionnaire included sociodemographic, sexual, behavioral and facility factor questions. Sociodemographic variables included age, sociocultural, orphan status and socioeconomic status. The variables in the sexual behavior section included age at sexual debut, number of sexual partners in the last 12 months, type of current partner, disclosure of HIV status, partners’ HIV status, history of sexually transmitted infections and contraception use. The facility-level questions focused on privacy for services, physical access, the attitude of the health workers and the availability of commodities.

Regarding the key informant sessions for the 12 healthcare providers, they were conducted by the lead author with prior experience of conducting interviews. The individual face-to-face interviews took place in a quiet place of the selected HIV clinic and written informed consent was obtained before participation. This also involved obtaining consent for audio recordings to be done. The interviews lasted 30–40 minutes each and were conducted in English. It took 4 weeks to sample and conduct the interviews due to the variation in availability of the health care workers. The interview guides had open-ended questions that focused on healthcare workers’ perception on sexual reproductive services utilization and associated factors among the youth living with HIV. Data saturation concluded interview sessions.

### Data processing and data analysis

Quantitative data was exported to data analytical software Stata version 14. Data cleaning and data coding was done. Descriptive statistics was used to describe the respondent baseline data, including sociodemographic profile information, sexual behavioral profile information, and level of sexual and reproductive utilization validated by the type of service used. The outcome variable utilization was coded “non-utilization” if one used 0–1 service in the last 6 months and “utilization” if one used the service more than once in the last 6 months. This was adapted from a study by Akerman *et al.* (2016).

Since the outcome variable was binary in nature, binary logistic regression was used to determine the association between the independent variables and dependent variable. Independent variables that were statistically significant at 95% confidence in univariate analysis and p value of less than 0.05 were taken for multivariable step wise regression analysis.

For qualitative data, audio recordings were transcribed verbatim to generate transcripts. NVivo version 14 was used to thematically analyze the transcripts. The responses from the open-ended questionnaires were analyzed using thematic analysis in NVivo version 14. The lead author carried out the generation of the themes and codes under supervision of the co-authors who are more experienced. The findings were presented with supporting quotes from the interviews.

### Ethical clearance and consent statement

Ethical approval and a research permit were sought before the start of this study. The ethical clearance to conduct the study was obtained from the ethical review committee of the Jomo Kenyatta University of Agriculture and Technology ethical review committee (JKU/2/41896B) on 25 ^th^ April 2023. A research permit was sought and obtained from the National Commission for Science Technology and Innovations (NACOSTI/P/23/25733) on 11 ^th^ May 2023. Nairobi county approval (NCCG/FHS/REC/364) and respective sub-county administrative approvals were obtained on 19 ^th^ May 2024. Administrative approvals from the respective hospitals for recruiting the study participants were obtained as well. All participants aged 18 years of age and above provided written informed consent before participating. For participants below 18 years old, written informed consent from their parents and assent from the participants were obtained.

Healthcare workers who participated in the individual key informant sessions provided written informed consent before participating. The informed consent process ensured full disclosure of study objectives, voluntary participation, study risks and benefits as well as results dissemination. To ensure privacy, data was stored in a password-protected drive accessible only to the lead author.

## Results

### The sociodemographic characteristics of the participants

253 participants were interviewed for this study. All questions were filled and answered. This demonstrated 100 % response rate. The Table 1 shows the sociodemographic of the respondents.

**Table 1.** Sociodemographic characteristics of participants.

Variables	Category	Frequency	Percentage %
Age category	15–19 years	86	33.99
	20–24 years	167	66.01
Sex	Male	79	31.23
	Female	174	68.77
Marital status	Single	206	81.42
	Married	37	14.62
	Separated	10	3.95
Education status attained	Never been to school	28	11.07
	Primary	26	10.28
	Secondary	117	46.25
	Tertiary	75	32.24
Current school status	No	139	54.94
	Yes	114	45.06
Religion	Catholic	96	37.94
	Muslim	36	14.23
	Protestant	105	41.5
	Traditional	8	3.16
	No religion	8	3.16
Employment status of parents	No	100	39.53
	Yes	153	60.47
Occupation of parents	Formal employment	35	22.88
	Casual labourer	37	24.18
	Self-employment/business	69	45.1
	Farmer	9	5.88
	Other	3	1.96
Employment Status of Respondents	No	163	64.43
	Yes	90	35.57
Occupation of respondents	Formal employment	22	24.44
	Casual laborer	36	40
	Self-employment/business	31	34.44
	Farmer	1	1.11

The ages of the participants ranged from 15–24 with a mean of 20 (Standard deviation 2.7). The majority (66.01%) of the participants were in the 20–24 age group. The majority (68%) of the participants were female. Out of 253 participants, 81.4% (206) were single, 14.6% were married and 3.95% were separated.

### The sexual behavioural profile of the participants

Table 2 shows the sexual behavioral profile of the participants. The mean sexual debut age of the respondent is 17.51 (Standard deviation 2.25). The majority (91.70%) of the participants have ever had sexual relationship. Out of 232 sexually active participants, 69.40% had one partner in the last 12 months, 18.10% had two partners, 10.34% had more than two partners, and 2.16% of the sexually active had zero partners in the last 12 months.

**Table 2.** Sexual behavioural profile of the respondents aged 15–24 years old living with HIV, Nairobi Kenya.

Variables	Category	Frequency	Percentage %
Ever had sexual relationship	No	21	8.30
	Yes	232	91.70
Number of sexual partners in last 12 months	One	166	71.55
	Two	42	18.10
	More than two	24	10.34
Type of current partner	Casual	206	81.42
	Regular	37	14.62
	Married	10	3.95
Disclosure of HIV status	Partner knows	92	39.66
	Partner doesn’t know	140	60.34
Partner ‘s HIV status	Unknown	139	59.91
	Positive	41	17.67
	Negative	52	22.41
HIV transmission concerns	Yes	52	22.41
	No	180	77.59
Sexually transmitted infections in last 12 months	No	203	87.50
	Yes	29	12.50
Obtained treatment for above STIs	Yes	19	65.52
	No	10	34.48
Pregnancy history	Previous pregnancy	45	19.40
	No previous pregnancy	139	59.91
	Made someone pregnant	8	3.45
	Never made anyone pregnant	40	17.24

At the time of the interview, the majority (81.42%) stated they had a casual partner, 14.62% had a regular partner, and 3.95% were married to their partners. On disclosure of HIV status, 54.74% of the respondents stated their partners are not aware of their status. 42.24% stated they had disclosed their HIV status to their partners while 7 respondents (3.02%) did not have a partner at the time of the interview.

When it came to knowing the HIV status of the partner, 133 (58.85%) of the respondents stated partner’s status was unknown, 23.01% stated partner’s status was negative and 18.14 % knew of positive HIV status of their partners.

Participants were asked if they have HIV transmission concerns; 77.59% of the participants reported they had no transmission concerns while 22.41% had HIV transmission concerns. Regarding sexually transmitted infections in the last 12 months, 12.50% had an infection while 203 (87.50%) did not have an infection.

### Utilization of sexual reproductive services

The outcome variable utilization was coded non utilization if one used 0–1 services in the last 6 months and utilization if one used more than 1 in the last 6 months.

52.96 % (134) of the participants did not utilize the services while 119 (47.04%) utilized the services. Table 3 shows the same information below.

**Table 3.** Level of utilization among participants aged 15–24 living with HIV in Nairobi Kenya.

Variables	Category	Frequency	Percentage
Utilization	non utilization	134	52.96
	utilization	119	47.04

***The types of sexual and reproductive services utilized***

**Figure 1.**
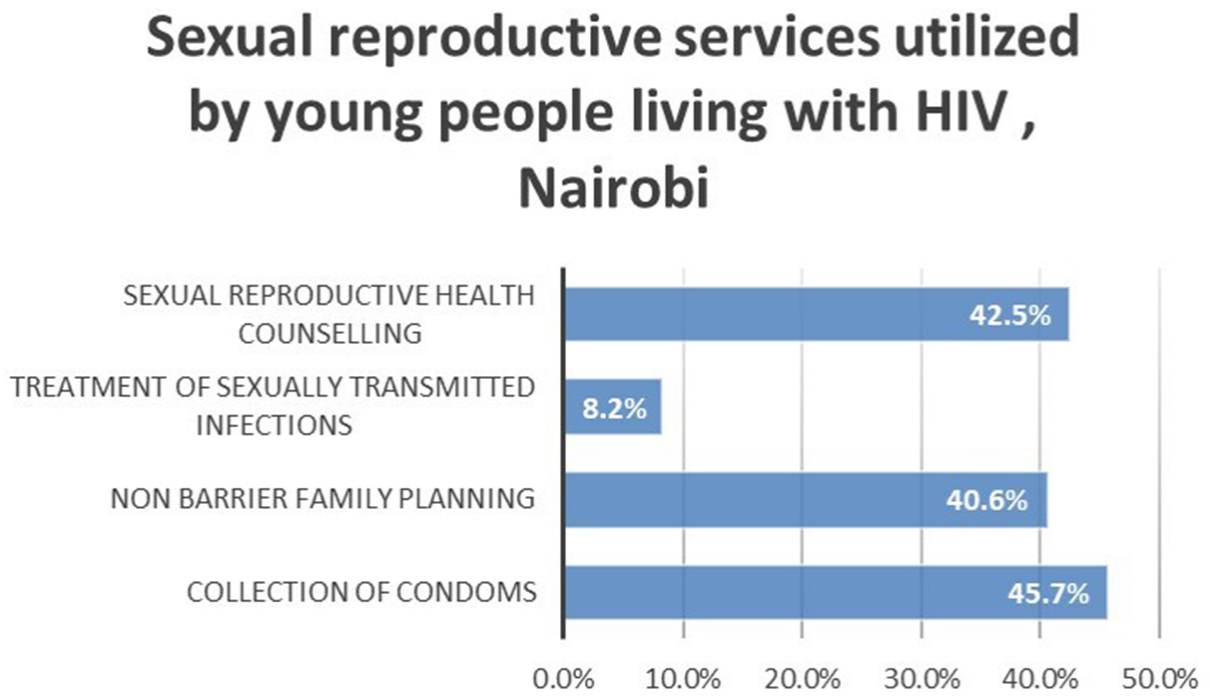
Sexual reproductive services utilized by young people living with HIV, Nairobi.

The utilization of sexual and reproductive services was validated by the type of sexual and reproductive services used. Sexual and reproductive health counselling services (42.5%) and collection of condoms (45.7%) were the most utilized while treatment of sexually transmitted infections (8.2%) was the least utilized. This is represented by Figure 1.

***Factors associated with the Utilization of Sexual Reproductive Health Services***

We examined the relationship of socio-demographic factors, sexual behavioral and facility related factors. In Table 4, factors used in the stepwise logistic regression are presented.

**Table 4.** Analysis of factors associated with the Utilization of Sexual Reproductive Health Services among young people living with HIV aged 15–24 years in Nairobi, Kenya (Stepwise binary logistic regression, best fit model).

Variables	Category	COR (95%Cl)	AOR	P value
Sex	Male	ref	ref	
	Female	2.50 (1.45-4.44)	3.26(1.67-6.40)	< 0.01 *
Age groups	20–24 years	3.40(1.94-5.97)	2.27(1.11-4.65)	0.03 *
	15–19 years	ref		
Currently in school	No	ref	-	
	Yes	1.10(.60-2.024)		
Marriage status	Married	3.16(1.48-6.75)	-	
	Single	Ref		
	Separated	1.34(0.38-4.77)		
Disclosure of HIV status	Partner knows	2.78(1.61-4.81)	2.00(1.11-3.80)	**0.04** *
	Partner doesn’t know	ref	ref	
HIV transmission concerns	Yes	2.78(1.60-4.81)	-	
	No	ref		
The facilities are conveniently located and available	Strongly agree/ Agree	5.41(2.32-12.60)	1.16(0.65-5.22)	0.80
	Neutral	2.00(0.79-5.08)	1.20(0.36-3.83)	0.80
	Strongly disagree / disagree	ref		
The sexual reproductive health services are in private	Strongly agree Agree	5.87(3.12-11.54)	3.27(1.42-7.60)	**0.01** *
	Disagree/strongly Disagree	Ref		
	Neutral	1.67(0.78-3.60)	1.40(0.98-5.60)	0.48
The staff have a welcoming attitude towards youth seeking sexual reproductive services	Strongly agree/ Agree	3.5(1.45-8.47)	2.35(0.83-6.60)	0.11
	Disagree/strongly disagree	Ref		
	Neutral	1.25(0.49-3.20)	1.40(0.44-4.41)	0.57
Commodities for sexual reproductive services are Available	Strongly agree/ Agree	2.70(1.41-5.20)	2.20(0.76-6.38)	0.05
	Neutral	0.90(0.40-1.94)	2.34(0.98-5.60)	0.14
	Strongly disagree / disagree	ref		

*Notes: * - statistically significant at 5% level (p<0.05) , COR -Crude Odds Ratio, ADR- Adjusted Odds Ratio

Female sex (AOR: 3.26 95%Cl: 1.67-6.40), increase in age (AOR: 2.30 95%Cl: 1.11-4.65), HIV status disclosure to a sexual partner (AOR: 2.00 95%Cl: 1.11-3.80) and privacy for sexual reproductive health services at a health facility (AOR: 3.27 95%Cl: 1.42-7.60) were factors significantly associated with utilization in multivariate regression. This is evident in Table 4.

Older youth are more likely compared to younger youth to utilize sexual and reproductive services (Crude odds ratio 3.4 AOR: 2.30, p value = 0.03). Married young people are 3 times more likely to utilize sexual and reproductive health services compared to their single youth peers (Crude odds ratio 3.16, p value < 0.01). Female youth have 3.6 higher odds of utilizing sexual and reproductive services compared to male youth (Crude odds Ratio 2.5, AOR: 3.26 p value < 0.01).

Youth who disclosed their HIV status to their sexual partner have twice as high odds of utilizing sexual and reproductive services compared to the youth who did not disclose their HIV status. (Crude odds Ratio 2.78 AOR: 2.00, p-value = 0.04).

Youth who positively felt that the facilities offer sexual and reproductive services in private are likely to utilize the services compared to those who negatively felt about the privacy (Crude Odds ratio 5.87 AOR: 3.27. p-value = 0.01).

### Qualitative Results

To triangulate the quantitative results, key qualitative results from key interviews and open-ended questions were summarized into themes, perceived ineligibility due to sociodemographic status, fear of disclosure of HIV status to sexual partner, lack of sexual risk awareness and health care workers attitudes.

**Perceived ineligibility due to sociodemographic status**

 Most participants expressed an internal belief on the sociodemographic prolife of the people who can utilize the sexual reproductive services. It was expressed among the young participants that sexual and reproductive services were for married people and females only.

“ *Am not married to seek these reproductive services*” Participant dh001

“ *Most services are targeted for females*” – Participant dh034

“ *… girls come in more often than boys... girls even come in for condoms for their boyfriends…” key informant interview 03*

**Fear of disclosure of HIV status to sexual partner**

The health care workers also stated that failure in disclosure of HIV status to the sexual partner is regarded as barrier to accessing sexual and reproductive health services.

“ *There is fear of disclosure among the young people and mingling with the opposite sex because they do not wish for their partners to know if they are infected so they wouldn’t come for the services together” – Key informant interview 07*

**Lack of sexual risk awareness**

Health care workers noted that there was a lack of safety transmission concerns among the young people living with HIV and the participants too reported that they had no sexual health concerns

“ *There are no sexual and reproductive concerns” – Participant ML005*

“I *[do] not [have] any concerns in the last 6 months” – Participant ML019*

“There *is fear of disclosure among the young people and mingling with the opposite sex because they do not wish for their partners to know if they are infected so they wouldn’t come for the services together” – Key informant interview 07*

**Health care workers attitudes**

The findings from the key informant interview also support that the attitude of the health care workers affecting the utilization of sexual and reproductive health services.

“Initially, *there was a lot of negativities especially for the adolescents to get the services but with continuous mentorship and health education there is much improvement among the health care providers, and I can say there is a lot of positivity*” - Key informant 05

Statements from the open-ended questions also have consistent findings on the same. In relation to the health care workers‘ attitudes, lack of privacy was among the reasons the young people living with HIV did not access the services.

*“I do not use it because there is no space designed for youth in the facility to access the services”*

*- Participant PM024*

The key informants stated the presence or absence of commodities did influence the utilization of sexual and reproductive services among young people living with HIV. They also stated this problem is not a persistent problem.

“Of *course, there have been stockouts… That’s what hinders services…. for instance, one wants to come for the family planning services… the implants are not available, so they go home unattended to. But it happens occasionally… on and off.*” *- Key informant 03*

## Discussion

This study aimed at determining factors associated with sexual and reproductive service utilization among young people living with HIV. This is an essential component in the health agenda for combating HIV transmission. Only 47.04% of young people living with HIV had utilization where they used one or more services in the last 12 months. This is below the expected standard given that these young people are in frequent contact with the health care system. A study in Ethiopia done by Motuma *et al.* (2016) had higher rates of utilization (64%). Studies in western Kenya by Embleton *et al.* (2023) and Abdurahman *et al.* (2022) had lower rates (36%) and 23% compared to our findings. This variation could be attributed to different settings of the study area as the health system. Other possible reasons could be differences in baseline respondents' backgrounds and time references used in the definition of SRH service utilization.

A variety of studies used different types or mixes of services to define youth-friendly sexual and reproductive services. The World Health Organization's definition of youth-friendly services includes family planning, voluntary counselling and treatment of sexually transmitted infections. This study did not examine voluntary testing as the study population was young people living with HIV. A cross-sectional study done by Amaje *et al.* (2022) had more components of sexual and reproductive services than this study; they used 8 components. The study however had similar ratings of the first three services compared to our study findings: sexual and reproductive health counselling (59.40%), condom collection (55.70%) and family planning (51.40%).

Age was found to be a significant sociodemographic factor associated with utilization among young people living with HIV based on results from this study. Older youth (above 18 years of age) are more likely to utilize the services compared to their younger peers (below 18 years of age). This finding is similar to results from a mixed-method cross-sectional study done in India by Banerjee *et al.* (2023) and another conducted in Kenya ( Nyang’echi & Osero, 2024). This phenomena might be attributed to increased level of autonomy among older youth and reduced dependency on care givers/parents hence can access the services at free will. In Sub-Saharan settings most youth (below 18 years) may require parental involvement to access health care services. This might delay or hinder utilization of sexual reproductive services.

Females are more likely to have higher utilization rates compared to males (AOR: 3.30 95% Cl: 1.67-6.40). This might be due to the perceived consequences females face such as unplanned pregnancy. These findings are similar to the results of the previous cross-sectional studies done in Ethiopia and a mixed method study in Nigeria ( Odo *et al*., 2021; Zepro *et al.*, 2023). This emphasizes the need for addressing sex specific needs according to different settings.

Though not in the final model, marital status is a sociodemographic variable worth noting. Married youth were more likely to utilize the services compared to those who were not married (COR:3.16 95% CL 1.48-6.75). Qualitative data from the opened questions also converged with these findings. Qualitative data from a study in Nigeria had consistent findings as well: it was a cultural belief that these services are taboo for unmarried people ( Odo *et al*., 2021). Married youth might be more culturally accepted to seek these services in society as their sexual activity is socially appropriate. This could be in contrast to the unmarried youth who might be facing judgement and stigma when seeking activities associated with sexual activity. Designing the services in a way that appreciates the unique characteristics of unmarried people is essential.

Among the sexual behavioral factors, HIV status disclosure to a sexual partner was a statically significant factor in the final model (AOR: 2.00 95% Cl: 1.11-3.80). Those who disclosed their status to their sexual partner have higher odds of utilizing the services. A study in South Africa conducted by Mengwai *et al.* (2020) reported disclosure to sexual partners as a predictor of sexual services utilization. Studies in Ethiopia and Kenya have similar findings with this ( Mulongo *et al.*, 2017; Tewabe *et al.*, 2020). This highlights that open disclosure of HIV status facilitates open communication about sexual and reproductive health and the use of its services. However, this is in contrast to a previous study done in Ethiopia ( Berhane *et al*., 2013). More than 50% of YPLWH reported low levels of HIV status disclosure to their sexual partners. Furthermore, many of them are not aware of their partner’s HIV status either ( Ndongmo *et al*., 2017).

There is an association between facility-level factors and the utilization of sexual and reproductive health services. Health facility-level factors such as the attitude of the health care providers, privacy and availability of commodities were found to be predictors of the use of the services in both qualitative and quantitative findings. This is in convergence with information from a previous study from Nigeria where the qualitative data stated major barriers to utilization include the attitude of the health care providers and lack of privacy ( Odo *et al.*, 2021). There is need in strengthening of partnerships with relevant stakeholders involved in pharmaceutical supply chain to ensure consistent supply of commodities.

The attitude of healthcare workers is an important determinant that influences the utilization of youth-friendly sexual and reproductive services ( Pettitt *et al*., 2013). This supports our findings in quantitative and qualitative arms. A mixed-method study in Kenya by Mulongo *et al*. (2017) stated negative attitudes of the health workers toward young people living with HIV limiting access to services. There have been several qualitative studies have pointed out that youth have negative perceptions of health facilities due to judgmental attitudes of the health workers offering the services. A study in Kenya and Zambia demonstrated that reproductive health services are underutilized due to negative attitudes as well as a lack of competence in the provision of services catered to the youth ( Warenius *et al*., 2006). This could be due to intrapersonal beliefs and poor professional skills in handling the sensitivity of sexual health topics among young vulnerable populations.

Privacy concern is a key barrier to the utilization of sexual services. This was found in quantitative results that those who had positive attitudes that privacy was meant were more likely to utilize the services compared to those who had neutral and negative attitudes. Poor privacy setting and practices is a maker of poor-quality services ( Robert *et al.*, 2020). Previous studies in Uganda had similar findings to this study and reported service features that threaten privacy such as poor infrastructure of the health facility ( Akatukwasa *et al*., 2019; Nalwadda *et al.*, 2016).

### Public Health Implications

The study findings highlight the need to conceptualize and implement interventions that will focus on improving utilization of sexual reproductive services among young people living with HIV.

Public health promotion activities that focus on breaking the socio-cultural beliefs that limit male youth from using the services. For instance, engagement of male youth champions on peer outreach activities regarding sexual reproductive health can be used to shit the masculine norms in society.

To improve HIV status disclosure among youth living with HIV to their sexual partners, implementation of behavioral interventions such as peer led support groups can be explored to empower safe disclosure. This in return can lead to better utilization rates of related sexual reproductive services.

Our findings on the compromised privacy due to structural infrastructure iterate the need for the government and relevant stakeholders to invent in set up of youth friendly facilities to address this barrier. Health care providers can receive training workshops on confidential handling of this vulnerable population in clinic. Stakeholders also need to reconsider the reorganization of health facilities to promote privacy by extending operating hours, improving waiting and consultation areas.

This study show cased that general population of young people has been the focus of previous sexual reproductive health studies. Future studies should tailor the study population to include vulnerable subpopulations such as young mothers living with HIV. Future studies should also incorporate strategies such delayed interviews, surrogate consent and medical record review to include critically ill youth (temporarily incapacitated individuals) in their research.

As this study was conducted in government health facilities, future research could focus on comparison of youth friendly services delivery between private health facilities and government health facilities.

### Study limitations

The study encountered some limitations. Firstly, due to age range and prejudices associated with HIV status, this group may have perceived social stigma which influenced their desire to disclose sensitive information on sexual activity data therefore this could have led to underreporting.

 Interview bias was minimized through standard training of data collectors, use of structured interview and supervision of coauthors to ensure neutrality and consistency.

The school season also affected the distribution of the participants as many youths were in boarding school at the time the data collection was being conducted. The target population was from Nairobi County therefore generalization to the other counties of Kenya may not be possible.

Regarding qualitative data, the audio files were transcribed and coded by the lead researcher. Regular review and feedback from the senior co-authors helped mitigate bias. This strengthened the credibility of the themes.

## Conclusion

53% of the participants had utilized services in the last 12 months at the time of the data collection. Those who were female, mature in age and had disclosed their HIV status to their sexual partners were more likely to utilize the services compared to their peers. Facility factors like commodity availability, privacy and attitude of the health care workers are important determinants as well. Though this vulnerable subpopulation has more frequent contact with health care service providers, the utilization of sexual and reproductive services is suboptimal, and it can be improved. Stakeholders are recommended to put more emphasis on strengthening behavioral interventions that promote male young men involvement and disclosure support strategies can address the existing disparities HIV disclosure to sexual partners.

## List of abbreviations

**Table:** 

HIV	Human immuno-deficiency virus
ARV	Antiretroviral therapy
STI	Sexually transmitted infections
SRH	Sexual and reproductive health
YPLWH	Young people living with HIV
WHO	World Health Organization

## Data Availability

Figshare: Utilization of Sexual Reproductive Health Services Among Young People Living with HIV attending Selected HIV clinics in selected sub counties of Nairobi, Kenya. https://doi.org/10.6084/m9.figshare.25880419 ( Phiri, 2024) The underlying data contains anonymized raw data file (in both DTA and excel formats) log file that contains codebook and regression multivariate model (DTA and PDF formats) Figshare: Utilization of Sexual Reproductive Health Services Among Young People Living with HIV attending Selected HIV clinics in selected sub counties of Nairobi, Kenya. https://doi.org/10.6084/m9.figshare.25880419 ( Phiri, 2024) The project contains the following extended data: Questionnaire Key informant interview guide Data is available under the terms of the Creative Commons Attribution 4.0 International License (CC-BY 4.0).
