# Supplementary material for: Utilization of sexual and reproductive health services among young people living with HIV and attending selected HIV clinics in selected sub-counties of Nairobi, Kenya
Source: Open Res Eur. 2025 Jul 25;4:134. Originally published 2024 Jul 3. [Version 2] doi: 10.12688/openreseurope.17611.2 (PMC12411835; doi:10.12688/openreseurope.17611.2)
Supplement: Peer review history

# Reviewer report: Reviewer response for version 2

**DOI:** 10.21956/openreseurope.22666.r57246

**Published:** 2025-09-04

**Authors:** Naomi Wekwete

**Affiliations:** 1 Department of Demography Settement and Development, University of Zimbabwe, Harare, Zimbabwe

**Overall Comments**

 This study makes an important contribution to understanding sexual and reproductive health (SRH) service utilization among young people living with HIV, who face the dual challenge of navigating adolescent health needs while managing HIV. The paper provides practical solutions, highlights the need to conceptualize and implement interventions to improve utilization of SRH services among adolescents living with HIV. however, there are several areas for improvement, outlined below.

**Abstract**

 You do not start a sentence with a number. It should be “Twelve …” (instead of “12 …”). General rule: In formal academic writing, numbers one to nine are usually written in words, while 10 and above are written as numerals unless they start a sentence—in which case they must also be written in words.

 “4Stepwise multivariable binary logistic regression was used to analyse the quantitative data using Stata version 14. NVivo software was used to code and thematically analyse the data.” Remove the stray “4” before Stepwise. Also, these statements do not fall under results but rather belong in Methods, not Results. Your Results section should simply report the findings (e.g., odds ratios, CIs) without restating the analytic tools.

 “Results 47 % of the participants had utilized the services”.  Delete “Results” and write 47% in words. Remove any extra spaces before the percentage sign (e.g., change 47 % → 47%). Ensure all percentages follow the same style consistently.

 Replace all instances of “sexual reproductive health” with the correct term “sexual and reproductive health” throughout the document to ensure consistency and accuracy.

**Introduction**

 Page 4, paragraph 6, line 7 – delete “done” and replace with ‘conducted”.

 Page 4, paragraph 6 – wrong citation – delete “Center for Reproductive **health**, 2021” and replace with “Center for Reproductive **Rights**, 2021”

 Page 4, paragraph 7, line 7 – delete “done” and replace with “conducted”.

 Page 5, paragraph 1, line 1 – the sentence “The drawback of this study is that the focus on the general population of the youth” is incomplete. It is not reading.

 Page 5, paragraph 2, line 1 – when presenting a number with more than three digits, use a separator, e.g., the number “131780” should read as “131,780”. In some sections, there is a separator for the number. Be consistent and correct the rest of the document where such numbers without a separator exist.

 Page 5, paragraph 2, line 8 – insert a full-stop after “Kenya”.

 Page 5, paragraph 3, line 1-4 – rephrase sentence which reads “data collection and analysis were investigated on utilization of …”. Data collection and analysis are not investigated. Consider: “data were collected and analyzed to examine the utilization of ….”  

**Methods and Materials**

 Insert a hyphen in all compound adjectives for readability, for example, change ‘facility based’ to ‘facility-based’ and ‘facility related’ to ‘facility-related’. Apply this consistently throughout the document.”

 Sampling techniques – Level 3 is presented in numeric form, while Levels Two and Four are written in words.” The general rule is that numbers one to nine are usually written in words, while 10 and above are written as numerals unless they start a sentence, in which case they must also be written in words.

 Participants – “They must provide signed informed consent for those above 18 years or provide written parental consent and assent for those below 18 years”. This was already done. So, it should be written in the past tense. Refer to the 12 participants as key informants, not as 12 individual key informant interview sessions. Delete the “s” on interviews.

 “Purposive sampling techniques were used to select 12 health care providers as key informants that are involved in treatments and managers of the six selected comprehensive care clinics (HIV clinics) in the six selected sub counties.” – This should fall under sampling techniques. Insert a hyphen between “sub counties” to “sub-counties” for readability. Also, standardize all instances of “sub counties” - “sub-counties” across the document.

 When conducting the quantitative, what language was used? It’s stated that “The interviews were conducted in English and Kiswahili.” Why have both languages, and under what circumstances was English used instead of Swahili?

 State why the English language was used in the key informant interviews.  

 Data processing and data analysis – a questionnaire is used on quantitative data only, not qualitative data. For qualitative data, consider phrasing such as: “The responses from the key informant interview questions were ….”.

**Results**

 Insert a heading “Quantitative Results”.

**“**253 participants were interviewed for this study.” - As mentioned above, the number 253 should be written in words when it starts a sentence, i.e., “ *Two hundred and fifty-three* participants were interviewed for this study”.

 “The Table 1 shows the sociodemographic of the respondents.” - Delete “ *The*” at the beginning of the sentence, insert “ *characteristics*” after “sociodemographic”.

 Single is not a marital status. Replace “ *single*” with “ *never married*”.

 The sentence “The mean sexual debut age of the respondent *is *17.51.” - Results should be presented in the past tense. Thus, it should be written as “The mean sexual debut age of the respondent *was* 17.51.” Maintain past tense in reporting results in the rest of the findings section.

 “At the time of the interview, the majority (81.42%) stated they had a casual partner, 14.62% had a regular partner, and 3.95% were married to their partners.” – replace “ *stated*” with “ *reported that*”.  

 “Out of 232 sexually active participants, 69.40% had one partner in the last 12 months, 18.10% had two partners, 10.34% had more than two partners, and 2.16% of the sexually active had zero partners in the last 12 months.” – replace “ *had one partner*” with “ *reported having one partner*” and replace “ *had zero partners*” with “ *reported having no partners*”.

 “.. 54.74% of the respondents stated their partners *are* not aware of their status.” – report in past tense. Correct the section of the results where findings are presented in the present tense.

 “When it came to knowing the HIV status of the partner, 133 (58.85%) of the respondents stated partner’s status was unknown, 23.01% stated partner’s status was negative and 18.14 % knew of positive HIV status of their partners.” – Replace “ *When it came to knowing* …” with “ *Regarding knowledge of* ….”

 Remove the full stop at the end of all section and table titles, and ensure this formatting is applied consistently throughout the document.

 “Table 3 shows the same information *below*.” – delete “ *below*”.

 Factors associated with – delete the sentence “ *This is evident in Table 4*” since Table 4 has already been cited earlier.

 “Older youth are more likely compared to younger youth to utilize sexual and reproductive services …”, “Married young people are 3 times more likely to utilize” – write in past tense. Maintain past tense in reporting results.

 Youth who disclosed their HIV status to their sexual partner have twice as high odds of utilizing sexual and reproductive services compared to the youth who did not disclose their HIV status. (Crude odds Ratio 2.78 AOR: 2.00, p-value = 0.04). - Delete the full stop after HIV status.

***Qualitative Results***

 Remove the italics on the qualitative results section from “key informant interview 03”, “Participant ML005”, “Participant ML019”, “Key informant interview 07”, etc., to ensure consistent formatting throughout the document.

**Discussion**

 The statement “Only 47.04%  of young people living with HIV had utilization where they used one or more services in the last 12 months”. Delete “only”.

 A study in Ethiopia done by Motuma et al. (2016) had higher rates of utilization (64%). - Delete the word “done” when referring to studies. Avoid using informal expressions like “done”, instead, use “by [author]”, “conducted by”, or “reported by” for formal academic writing.

 Ensure consistent tense usage throughout the Discussion section. When reporting study findings, use the past tense (e.g., ‘Married youth were more likely to utilize the services’). Use the present tense only when interpreting or discussing the implications of the results (e.g., ‘These findings highlight the need for targeted interventions’). Avoid switching tenses arbitrarily within the same context.”

 “Qualitative data from the opened questions” – replace “ *opened*” with “ *open-ended*”

 On the statement: “There have been several qualitative studies have pointed out that youth have negative perceptions of health facilities due to judgmental attitudes of the health workers offering the services”. Insert “that” after “qualitative studies …”.

  Grammatical error -  “This was found in quantitative results that those who had positive attitudes that privacy was meant were more likely to utilize the services compared to those who had neutral and negative attitudes.” Not reading well.

**Public Health Implications**

 The statement: “For instance, engagement of male youth champions on peer outreach activities regarding sexual reproductive health can be used to shit the masculine norms in society.” Replace “sexual reproductive health” with “sexual and reproductive”, “ *shit*” with “ *shift*”.

**References**

 The author states that “According to the Open Europe journal guidelines, only research journal articles should be placed in the references. Citations from web articles, etc., only need to be hyperlinkinked”. From my field, all the citations should be included in the references, including citations from the web.

Is the study design appropriate and does the work have academic merit?

Yes

Is the work clearly and accurately presented and does it cite the current literature?

Yes

If applicable, is the statistical analysis and its interpretation appropriate?

Yes

Are all the source data underlying the results available to ensure full reproducibility?

Yes

Are the conclusions drawn adequately supported by the results?

Yes

Are sufficient details of methods and analysis provided to allow replication by others?

Yes

Reviewer Expertise:

Population studies - sexual and reproductive health, fertility, morbidity and mortality, and migration.

I confirm that I have read this submission and believe that I have an appropriate level of expertise to confirm that it is of an acceptable scientific standard.

# Reviewer report: Reviewer response for version 1

**DOI:** 10.21956/openreseurope.19030.r43738

**Published:** 2024-09-19

**Authors:** Melkamu Meried Mengesha

**Affiliations:** 1 Department of Health Sciences, Faculty of Medicine, Lund University, Lund, Sweden

The article presented important findings related to the SRH services utilization among youth living with HIV. It utilized mixed methods to complement findings from each method and provide thick description of the problem studied. However, the article could benefit from the comments, particularly with the selection of study participants and method of analysis employed.

 Abstract

 Results: " the most utilized while treatment of sexually transmitted infections (8.2%) was the least utilized services." Who is in the denominator for this figure, the total sample or total of those who had some STI?

 Expand abbreviation on first use, e.g., AOR--adjusted odds ratio.

 Female sex and disclosure of HIV status is associated with positive uptake. The recommendation for male partner involvement is not clear in the abstract with the above findings highlighted.

 Introduction:

 How is the context of SRH services uptake differ among young people living with and without HIV? Do they share a common factor? Presenting details on this could put the problem in context justifying the need of the current study its value to add.

 Inclusion: The authors presented that the involved 12 healthcare workers in the interview, but this was not included in the inclusion criterion.

 Exclusion criteria: "The youth that are too sick to answer questions at the time," authors reflection on the risk of selection bias with this criterion, particularly as they consider seeking treatment for STI as one of the SRH services uptake.

 sampling technique: please provide detailed descriptions separately for the quantitative and the qualitative parts with subheadings. And provide justification as to why adolescents the young people living with HIV are not included in the qualitative interview.

 sample size calculation: "Calculations was done using the prevalence of 32.8 % by a similar study in Ethiopia": prevalence of what is referred in here? please include text for clarification.  

 Data collection: "Four weeks were needed for recruitment and to collect data from participants in the month of June 2023." Did the authors conducted data collection as potential participants visit for service or how? Please give detailed explanation on how the data collectors managed to find study participants over the study period.

 Interview duration(s): there are two interview durations presented, but it is not clear which is for which method. Better again if the details are presented per the method of interview involved.

 Data processing and data analysis: As the authors utilized a multi-stage sampling, factors beyond the individual level could play a role in producing the outcome. Authors reflection is needed on whether the results could be different if they have used a multilevel binary logistic regression. "For qualitative data, audio recordings were transcribed verbatim to generate transcripts" please indicate the interview language used in the study context.

 Ethics: Who were the data collectors? how protected the study participants were in terms of their private information of HIV diagnosis? Please include details if how youth with STI and did not receive Rx are handled during the study.

Is the study design appropriate and does the work have academic merit?

Yes

Is the work clearly and accurately presented and does it cite the current literature?

Yes

If applicable, is the statistical analysis and its interpretation appropriate?

Partly

Are all the source data underlying the results available to ensure full reproducibility?

Yes

Are the conclusions drawn adequately supported by the results?

Yes

Are sufficient details of methods and analysis provided to allow replication by others?

Partly

Reviewer Expertise:

Epidemiology, Adolescent and HIV research.

I confirm that I have read this submission and believe that I have an appropriate level of expertise to confirm that it is of an acceptable scientific standard, however I have significant reservations, as outlined above.

# Response

**Published:** 2025-07-10

**Authors:** Nomsa Phiri

**Affiliations:** Environmental Health and Disease Control, Jomo Kenyatta University of Agriculture and Technology, Nairobi, Nairobi County, Kenya

1.the most utilized while treatment of sexually transmitted infections (8.2%) was the least utilized services." Who is in the denominator for this figure, the total sample or total of those who had some STI? *The total sample number interviewed is the denominator* 2.Expand abbreviation on first use, e.g., AOR--adjusted odds ratio. This has been done in the abstract paragraph 3 line 6

 Female sex and disclosure of HIV status is associated with positive uptake. The recommendation for male partner involvement is not clear in the abstract with the above findings highlighted. *Given  higher utilization of sexual reproductive services was demonstrated among females and those who disclosed their HIV status to sexual partner , strengthening behavioral  interventions to engage young men and disclosure support strategies can address the existing disparities* . This was modified in abstract section paragraph 4

**Introduction:** 3. How is the context of SRH services uptake differ among young people living with and without HIV? Do they share a common factor? Presenting details on this could put the problem in context justifying the need of the current study its value to add. *This has been addressed in the introduction  paragraph 2.* Inclusion: The authors presented that the involved 12 healthcare workers in the interview, but this was not included in the inclusion criterion. *T his has been added* .in methodology section paragraph 7 line 1

 4.Exclusion criteria: "The youth that are too sick to answer questions at the time," authors reflection on the risk of selection bias with this criterion, particularly as they consider seeking treatment for STI as one of the SRH services uptake. Thank you for the comment . We acknowledge that  excluding critical ill youth could have introduced slection bias  underestimates the true SRH for STI treatment .Due to study design , study location of outpatient and ethical guidelines , critically ill may lack capacity  to provide informed consent .We acknowledge this limitation  and we have made suggestions for future studies  to incorporate strategies ( delayed interviews , surrogate consent   and medical record review to include temporarily incapacitated individuals  in the public health implications sections  

 sampling technique: please provide detailed descriptions separately for the quantitative and the qualitative parts with subheadings. And provide justification as to why adolescents the young people living with HIV are not included in the qualitative interview. Sampling techniques have been separated . *Sampling techniques have been separated for quantitative and qualitative parts in the methodology section. The young people living with HIV were provided with a semi instructed questionnaire  . Responses to the open-ended questions in the questionnaires were written down and coded. Due to the sensitive nature of  topic and stigma surrounding discussion of sexual activity even among young people, providers who are in usual contact with them  were used to minimize social desirability bias* .

 sample size calculation: "Calculations was done using the prevalence of 32.8 % by a similar study in Ethiopia": prevalence of what is referred in here? please include text for clarification. *using the prevalence of 32.8 %  for utilization of sexual reproductive services by a similar study in Ethiopia ( Amaje et al., 2022.This is modified in methodology paragraph 3 line 111*

 5.Data collection: "Four weeks were needed for recruitment and to collect data from participants in the month of June 2023." Did the authors conducted data collection as potential participants visit for service or how?  Please give detailed explanation on how the data collectors managed to find study participants over the study period. *This has been added to the methodology paragraph 3 lines 105-108. Data collection was from youth coming in selected HIV clinics for scheduled visits for their ART refill.*

 Interview duration(s): there are two interview durations presented, but it is not clear which is for which method. Better again if the details are presented per the method of interview involved. *Four weeks were needed for recruitment and to collect data from participants in the month of June 2023 for both qualitative and quantitative methods. This has been clarified in the methodology paragraph 1 line 84*

 6.Data processing and data analysis: As the authors utilized a multi-stage sampling, factors beyond the individual level could play a role in producing the outcome. Authors reflection is needed on whether the results could be different if they have used a multilevel binary logistic regression. Thanks for your comment on appropriateness of the analytical method, particularly in the context of multi-stage sampling and the potential utility of multilevel binary logistic regression. We acknowledge that, ideally, a multilevel modeling approach could account for clustering effects at the facility level and better capture factors operating beyond the individual level. However, our study included only six health facilities, which limited the statistical feasibility and stability of fitting a reliable multilevel model. As is widely recommended in the literature, multilevel modeling typically requires a larger number of clusters (e.g., 20 or more) to yield valid and robust estimates. With only six clusters, there was a heightened risk of biased standard errors and unstable estimates. Furthermore, our study employed a mixed-methods design, integrating both quantitative and qualitative data to enhance the depth and contextual understanding of the findings. The qualitative component addressed the potential multi-level influences that may not have been captured through statistical modeling alone. The triangulation of the findings supports the credibility and comprehensiveness of our findings, even in the absence of multilevel regression. Given these considerations, we believe that the chosen method, a standard binary logistic regression, was appropriate and justified, considering both the sample structure and the broader mixed-methods framework of the study.”

*"For qualitative data, audio recordings were transcribed verbatim to generate transcripts" please indicate the interview language used in the study context.* *English was used for the key interviews ( qualitaitve arm).This has been in the methodology section*

 7. Ethics: Who were the data collectors? how protected the study participants were in terms of their private information of HIV diagnosis? Please include details if how youth with STI and did not receive Rx are handled during the study. *Regarding quantitative aspects, 6 research assistants with  previous experience of youth mentorship and data collection were recruited to serve as data collectors. Interviews were conducted in the private spaces of the same HIV clinics the participants attended. Filled questionnaires had identifiers removed. In this particular all youth had mentioned the history of STI had already received STI treatment.*

# Reviewer report: Reviewer response for version 1

**DOI:** 10.21956/openreseurope.19030.r43746

**Published:** 2024-09-11

**Authors:** Sylvia Ayieko

**Affiliations:** 1 Department of Health Promotion and Behavioral Sciences,, The University of Texas Health Science Center at Houston at School of Public Health, Houston, USA

General comments

 Overall, this is a well-written paper. This is a very important topic and critical for this specific population. I would encourage the authors to review the paper and include some active language for clarity. The methods and results should be organized into quantitative and qualitative sub-sections for better flow. The discussion needs to be strengthened by clarifying the findings. What do the authors think of the results? This needs to be expounded. Specific comments are outlined below.  

Specific comments

**Abstract**

 Consider revising the language for most of the abstract. Use active language for clarity. Who did what? What

1. The objective should be clear. For example, state…. “This study aims to…” or “The objective of this study is……”
2. It is not clear if the health care informants were just present in the key informant sessions or if they were the key informants. Were the sessions interviews of focus groups?
3. Mention that multivariable analysis was done

**Introduction**

1. The introduction is good, but it needs background information on the situation in Kenya, specifically in Nairobi County. Why focus on this specific population? What is the prevalence of HIV among youth in Kenya or Nairobi?
2. What are some examples of SRH services that are probably not being utilized well? Include a brief sentence or two.
3. Provide evidence for the high burden of HIV in Kenya—prevalence and mortality rates among adolescents. Emphasize the health issues in Kenya and Nairobi. There are quite a few studies on SRH among adolescents living with HIV/ AIDS in Kenya.  Include this in your introduction then highlight the gap.

**Methods**

1. What were the health facilities? Level 2, 3, 4, or 5? Just mentioning health facilities is vague.
2. As a mixed paper, it may help separating the methods for the quantitative and the methods for the qualitative study.
3. Were the interviews done in English or Swahili? Or was there an exclusion criterion based on language? Who translated/ transcribed the interviews? How many people coded the transcripts? Any coding done in the thematic analysis? How was consensus achieved?
4. Was it multivariate or multivariable analyses? (One outcome variable)

**Results**

1. The tables are good, but they are too many. I would recommend consolidating the tables to maybe 4 or 5. Results from the binary and multivariable regressions could be put in one table. Then, just include a  “-” where the analysis was not done.
2. For lower p-values, would recommend writing them as p < 0.01 rather than p= 0.00
3. Consider separating the quantitative results from the qualitative findings.
4. How many key informants were interviewed?
5.  Are there any specific themes from the qualitative findings?

**Discussion**

1. Elaborate on the findings. Why do you think age was a factor associated with utilization? First, provide possible explanations, then use evidence from other studies to support your results or explain any differences.
2. Discuss further why married youth were more likely to utilize the SRH services compared to their unmarried peers.
3. How does this study impact future research?
4. What should healthcare providers do to improve utilization? Expand on improvements in privacy, attitudes, and availability of SRH commodities  

***Limitations***

1. If only one person transcribed the qualitative data, there should be a discussion on how this may have influenced the data analysis.
2. Primary data should not be considered a limitation but actually a strength. The interviewer bias may be an issue but explain how this was resolved/addressed. 
3. Consider how this population may be facing stigma and the impact it could have on the study findings

**References**

1. Include all the citations in the references.
2. Quite a few are missing, including those from WHO, UNAIDS, and the Policies from Kenya.

Is the study design appropriate and does the work have academic merit?

Yes

Is the work clearly and accurately presented and does it cite the current literature?

Yes

If applicable, is the statistical analysis and its interpretation appropriate?

Partly

Are all the source data underlying the results available to ensure full reproducibility?

Yes

Are the conclusions drawn adequately supported by the results?

Partly

Are sufficient details of methods and analysis provided to allow replication by others?

Partly

Reviewer Expertise:

Adolescent health and sexual and reproductive health

I confirm that I have read this submission and believe that I have an appropriate level of expertise to confirm that it is of an acceptable scientific standard, however I have significant reservations, as outlined above.

# Response

**Published:** 2025-07-10

**Authors:** Nomsa Phiri

**Affiliations:** Environmental Health and Disease Control, Jomo Kenyatta University of Agriculture and Technology, Nairobi, Nairobi County, Kenya

1 **.Abstract** Consider revising the language for most of the abstract. Use active language for clarity. Who did what? What The objective should be clear. For example, state…. “This study aims to…” or “The objective of this study is……” *Thank you. The phrasing has been revised (  abstract paragraph 1 line 6)* 2.It is not clear if the health care informants were just present in the key informant sessions or if they were the key informants. Were the sessions interviews of focus groups? The health workers were the key informants. The key informant sessions were conducted at individual level ( abstract  paragraph 2 line 6) 3.Mention that multivariable analysis was done *Thank you. This was added.  (abstract paragraph 3 line 1)*

 4 **.Introduction** The introduction is good, but it needs background information on the situation in Kenya, specifically in Nairobi County. Why focus on this specific population? What is the prevalence of HIV among youth in Kenya or Nairobi? *detail has been provided in the introduction * 5.What are some examples of SRH services that are probably not being utilized well? Include a brief sentence or two. *Thank you .This has been added  in the introduction ( introduction  section paragraph 6 lines 5-7)*   6.Provide evidence for the high burden of HIV in Kenya—prevalence and mortality rates among adolescents. Emphasize the health issues in Kenya and Nairobi. There are quite a few studies on SRH among adolescents living with HIV/ AIDS in Kenya.  Include this in your introduction then highlight the gap. *Prevalence has been included in the introduction. Several studies conducted in kenya   have been included*

**Methods** 7.What were the health facilities? Level 2, 3, 4, or 5? Just mentioning health facilities is vague. *Majority of the facilities were level 3 . This has been added to the methodology*.(paragraph 3, lines 5-7 6.

 Provide evidence for the high burden of HIV in Kenya—prevalence and mortality rates among adolescents. Emphasize the health issues in Kenya and Nairobi. There are quite a few studies on SRH among adolescents living with HIV/ AIDS in Kenya. 

 Include this in your introduction then highlight the gap. *Prevalence has been included in the introduction. Several studies conducted in kenya   have been included*

**Methods** 7.What were the health facilities? Level 2, 3, 4, or 5? Just mentioning health facilities is vague. *Majority of the facilities were level 3 . This has been added to the methodology*.(paragraph 3, lines 5-7 8.As a mixed paper, it may help separating the methods for the quantitative and the methods for the qualitative study. *Thank you. Methodologies have been separated.* 9.Were the interviews done in English or Swahili? Or was there an exclusion criterion based on language? Who translated/ transcribed the interviews? How many people coded the transcripts? Any coding done in the thematic analysis? How was consensus achieved? *There was no exclusion criterion based on language .For  the quantitative , interviews were conducted in both English and Kiswahili. Interviews was conducted in English regarding the qualitative arm due to the higher education level of the participants for the key interviews. Lead author conducted the thematic analysis with supervision from co authors .Consensus was achieved through comparison with existing literature . This has been explained in the methods section paragraphs 5 , 7 and 10* 10. Was it multivariate or multivariable analyses? (One outcome variable) *Mutivariable analysis was conducted* .

**Results** 11.The tables are good, but they are too many. I would recommend consolidating the tables to maybe 4 or 5. Results from the binary and multivariable regressions could be put in one table. Then, just include a  “-” where the analysis was not done. This has been adapted. Only 4 tables are presented Bivariable and multivariable regression have merged into one table. 12.For lower p-values, would recommend writing them as p < 0.01 rather than p= 0.00 Consider separating the quantitative results from the qualitative findings. *Thank you. Quantitative and qualitative have been separated in the results section.* 13.How many key informants were interviewed? 12. This has been highlighted in abstract section paragraph 3 line 6 and methodology paragraph 7 line 140  Are there any specific themes from the qualitative findings? *This has been added in the results*

**Discussion** 14.Elaborate on the findings. Why do you think age was a factor associated with utilization? First, provide possible explanations, then use evidence from other studies to support your results or explain any differences. *Thank you. This has been modified  in the discussion section paragraph 3*

 15.Discuss further why married youth were more likely to utilize the SRH services compared to their unmarried peers. *This has been expanded in the discussion section paragraph*

 16.How does this study impact future research? *This has been  addressed under paragraphs 4 and 5  in public health implication section of the study*

 17.What should healthcare providers do to improve utilization? Expand on improvements in privacy, attitudes, and availability of SRH commodities *This has been addressed in the public health implications*

**Limitations** 18.If only one person transcribed the qualitative data, there should be a discussion on how this may have influenced the data analysis. *This has  been included in limitations section paragraph 4*

 19. Primary data should not be considered a limitation but actually a strength. The interviewer bias may be an issue but explain how this was resolved/addressed. *This has been explained in the limitation section  paragraph* 2 .

 20.Consider how this population may be facing stigma and the impact it could have on the study findings *This has been addressed in limitations section paragraph 1*

**References** 21.Include all the citations in the references. Quite a few are missing, including those from WHO, UNAIDS, and the Policies from Kenya. *According to the Open Europe journal guidelines, only research journal articles should be  placed in the references . Citations from web articles etc only need to be hyperlinkinked*
